# MiR-130a in the adipogenesis of human SGBS preadipocytes and its susceptibility to androgen regulation

**DOI:** 10.1080/21623945.2020.1750256

**Published:** 2020-04-09

**Authors:** Thomas Greither, Carina Wenzel, Julia Jansen, Matthias Kraus, Martin Wabitsch, Hermann M. Behre

**Affiliations:** a Center for Reproductive Medicine and Andrology, University Hospital Halle (Saale), Martin Luther University Halle-Wittenberg, Halle, Germany; b Division of Pediatric Endocrinology and Diabetes, Department of Pediatrics and Adolescent Medicine, Ulm University Medical Center, Ulm, Germany

**Keywords:** Adipogenesis, androgens, microRNA, miR-130a

## Abstract

**Objectives**: Adipogenesis is the differentiation process generating mature adipocytes from undifferentiated mesenchymal stem cells. The differentiation can be inhibited by androgens, although knowledge about intracellular effectors of this inhibition is scarce. Recently, androgen-regulated microRNAs were detected as interesting candidates in this context. In this study, we analyse the role of miR-130a and miR-301 in the adipogenesis of human SGBS preadipocytes and whether they are prone to androgen regulation. **Materials and Methods**: microRNA expression during adipogenic differentiation with or without androgen stimulation was measured by qPCR. Putative target genes of miR-130a and miR-301 were identified by target database search and validated in luciferase reporter assays. **Results**: miR-130a and miR-301 are both significantly downregulated on day 3 and day 5 of adipogenic differentiation in comparison to day 0. Under androgen stimulation, a significant upregulation of miR-130a was detected after 7 days of adipogenesis lasting to day 14, while miR-301 did not change significantly until day 14. Luciferase reporter assays revealed the androgen receptor (AR), adiponectin (ADIPOQ) and tumour necrosis factor alpha (TNFα) as miR-130a target genes. **Conclusions**: miR-130a is an androgen-regulated microRNA that is downregulated during the early phase of adipogenesis and exerts its functions by regulating AR and ADIPOQ translation. These data may help to identify new signalling pathways associated with the androgen-mediated inhibition of adipogenesis.

## Introduction

The differentiation process undergone by human mesenchymal stem cells to generate mature adipocytes is designated as adipogenesis, in contrary to the generation of myocytes (myogenesis) or osteocytes (osteogenesis) [1]. Adipogenesis is commonly divided into two phases: the determination phase, where the fate of the mesenchymal stem cell is determined, and the terminal differentiation process, where irreversible changes in the cell morphology as well as the cellular metabolism take place [2]. In the end, the mature adipocytic phenotype is developed by the massive uptake of triglycerides.

The adipogenic differentiation programme is tightly controlled by various molecular determinants, including the peroxisome proliferator activated receptor gamma (PPARγ) [3,4], the Wnt signalling pathway [5] and CCAAT/Enhancer binding proteins (CEBPs) [6]. Wnt signalling exerts mainly a suppression of mesenchymal stem cell differentiation towards adipocytes in favour of a differentiation towards osteoblasts [7,8], CEBPs and PPARγ act as key regulators for a plethora of protein determinants of adipogenic differentiation [4]. Both of these signalling pathways are heavily controlled by post-transcriptional silencing mechanisms during the differentiation process, mainly exerted by small non-coding microRNAs. While Wnt signalling is mainly suppressed in the initiation phase of adipogenesis, for instance by miR-204-5p [9], miR-148a-3p [10] or miR-199a-3p [11], translation repression of PPARγ has to be released for instance by downregulation of miR-27b [12] or miR-540 [13]. Furthermore, PPARγ can also activate the upregulation of microRNAs during the process of adipogenesis [14]. As an example, miR-378 is regulated alongside with PPARγ coactivator 1B (PGC1β) and promotes adipogenesis in subcutaneous, but not visceral fat tissue [15]. Taken together, microRNAs play a pivotal role in the orchestration of the different molecular determinants of the adipogenic process.

It is widely acknowledged that subnormal androgen serum levels in males (hypogonadism) are associated with various adverse physiological effects, including the accumulation of visceral fat mass frequently associated with the development of a metabolic syndrome and subsequent conditions like insulin resistance and diabetes mellitus type 2 or cardiovascular diseases [16–18]. Thus, the substitution of testosterone leads to some extent to the loss of fat mass and the increase of lean muscle mass in several clinical trials [19,20]. Furthermore, *in vitro* experiments demonstrated that androgens inhibited the adipogenic differentiation of murine preadipocytic cell lines [21,22] or human mesenchymal stem cells [23,24] in favour of myogenesis or osteogenesis. However, knowledge about the underlying molecular mechanisms in general and the involved, putatively androgen-regulated, microRNAs in special is scarce.

A recent report has demonstrated that among others miR-130a is specifically upregulated in differentiating osteoblasts, indicating a role in the shifting of the mesenchymal stem cell fate towards osteogenesis [25]. While human miR-130a is located on chromosome 11 as a single gene with own promoter, other family members bearing the same seed sequence like miR-130b and miR-301b are sharing a polycistronic cluster on chromosome 22. The role of miR-130a especially in the chemoresistance of tumourous diseases is extensively studied [26], as well as the function of miR-301 in the progression of several cancers [27,28]. On the other hand, less is known about the regulation of the miR-130a or miR-301 during adipogenesis.

Therefore, the aim of this study was (1) to evaluate the expression of miR-130a/301 during adipogenic differentiation w/o androgen stimulation and (2) to determine adipogenesis-related target genes for miR-130a.

## Material and methods

### Cell culture and adipogenic differentiation

Human SGBS cells are characterized as non-immortalized pre-adipocytic cell strain, and were kindly provided by Prof. M. Wabitsch (Ulm University Medical Centre, Germany). SAOS-2 cells (human osteosarcoma cell line) were purchased from DSMZ (Braunschweig, Germany), and cultured upon experimental usage in high glucose DMEM (Life technologies, Carlsbad, CA, USA) supplemented with 10% FBS (Life Technologies) and 100 U/l penicillin/streptomycin (Life technologies) at 5% CO_2_ and 37°C. Cell culture of SGBS cells and adipogenic differentiation induction were performed as described before [29]. Additional steroid treatment with testosterone (100 nM) or dihydrotestosterone (30 nM) was performed analogous to our previous work [30]. Briefly, adipogenic differentiation was initiated with ADM (adipogenic differentiation medium). Additionally, testosterone (100 nM) or dihydrotestosterone (30 nM) were added to the designated cell flasks, and the ADM ± steroids was replaced every 2 days in the first 4 days of treatment, and then on day 5, day 7 and day 10 of differentiation. Successful induction of adipogenesis was controlled in a test experiment beforehand by staining with 0.3% Oil Red O (Sigma-Aldrich, St. Louis, USA) after fixation in 10% paraformaldehyde (Sigma-Aldrich).

### RNA isolation

Phenol/chloroform extraction of total RNA was performed using TRIzol reagent (Life Technologies) according to the manufacturer’s protocol. Cells of the respective experiments were suspended by trypsin digestion (Life Technologies) and pelleted by centrifugation at 4000xg for 5 minutes. Cell pellets were dissolved in TRIzol reagent, mixed with chloroform (AppliChem, Darmstadt, Germany) and centrifuged at 10,000xg for 10 minutes at 4°C. Supernatant was collected, treated with DNase (Qiagen, Hilden, Germany) for 15 minutes to remove DNA contaminations, and then RNA was precipitated by the addition of 100% isopropanol (Applichem) overnight. RNA pellets were washed twice in 96% and 70% ethanol (Merck, Darmstadt, Germany) and dissolved after drying in 20 µl DEPC-H_2_O (Carl Roth, Karlsruhe, Germany). RNA concentrations and A_260/280_ ratio were determined by spectrographic analysis (biophotometer, Eppendorf, Hamburg, Germany).

### cDNA synthesis and quantitative real-time PCR

For quantitative real-time PCR (qPCR) measurements of the specific microRNAs, cDNA was synthesized using 10 ng of the respective total RNA samples by stem-loop RT-PCR from the TaqMan system (Thermo Fisher Scientific, Waltham, MA, USA; miR-130a: Assay #000454; miR-301: Assay #000528). RT reaction was initiated with Revert Aid reverse transcriptase kit (Thermo Fisher Scientific) according to the manufacturer’s instructions at 25°C for 10 min following 42°C for 1 hour (Thermocycler T3000, Biometra, Jena, Germany). qPCR was performed on a MyiQ iCycler (Biorad, Hercules, CA, USA) with commercially available TaqMan primer (Thermo Fisher Scientific). Relative miR-130a and miR-301 expression was calculated by 2^−ΔCT^ method, with U18 snoRNA (Thermo Fisher Scientific, Assay #001204) serving as normalization gene.

### Luciferase reporter assay

Putative miR-130a/301 binding sites in adipogenesis-related genes were identified by database search in TargetScan, PicTar and miRanda (see Table 1). The respective regions in the 3ʹUTRs of androgen receptor (AR), adiponectin (ADIPOQ), PPARγ, tumour necrosis factor alpha (TNFα), leptin, Ras dexamethasone-induced 1 (RASD1) and ARHGEF-12 were amplified by PCR (Qiagen, Hilden, Germany) with the respective primer (Sigma-Aldrich, see Table 2). Portions of the amplified sequences were used for mutagenesis of the respective microRNA binding sites (Agilent, Santa Clara, CA, USA). Both wildtype (wt) and mutated (mt) target gene 3ʹUTR amplificates were ligated to a psiCheck2 reporter plasmid (Promega, Madison, WI, USA) containing a firefly luciferase gene for normalization and a renilla luciferase gene for regulation detection. SAOS-2 cells were cultivated in 96 well plates (15,000 cells/cm^2^) and transfected with psiCheck2-reporter plasmids w/o 3ʹUTR inserts (Fugene, Promega). Additionally, cotransfection with miR-130a mimics (Ambion/Thermo Fisher Scientific) was performed with INTERFERin (PolyPlus Transfection, Illkirch, France) in some wells to identify miR-130a regulated 3ʹUTR sites. Transfected cells were cultivated for 24 h. Then, luciferase activities were tested by incubation of the cells with Dual-Glo Luciferase assay system (Promega), and subsequent luminescence read-out was measured on a luminescence plate reader (Tecan, Männedorf, Switzerland).

**Table 1.** miR-130a/miR-301 target genes predicted by several target gene databases. Numbers indicate Total Context ++ score (TargetScan) or PicTar Score (PicTar)

Gensymbol	TargetScan release 7.2 *	PicTar **	miRanda, release Aug 2010 ***
AR	−0.34		Predicted
ADIPOQ			Predicted
PPARγ	−0.6	4.28	Predicted
TNFα	−0.38		Predicted
Leptin			Predicted
RASD-1	−0.05	3.24	Predicted
ARHGEF-12	−0.24	1.63	Predicted

* http://www.targetscan.org/vert_72/

** https://pictar.mdc-berlin.de/

*** http://www.microrna.org/microrna/home.do

**Table 2.** Primer sequences used for 3ʹUTR amplification or mutagenesis

primer	Sequence
ADIPOQ-fw	5‘-CTCGAGAGCTCCTGTCTTGGAAGGACTAC-3‘
ADIPOQ-rv	5‘-GCGGCCGCAACCTGAAGTCTCAGCTACTC-3‘
AR-fw	5‘-CTCGAGAGCCCCAGTGAAGCATTGGAAAC-3‘
AR-rv	5‘-GCGGCCGCAGTGTGGCTGGCACAGAGTAG-3‘
ARHGEF12-fw 1	5‘-GGTGGCGCACTCGAGCCGAGGGTGGGATGGGAGTGGGTA-3‘
ARHGEF12-rv 1	5‘-GGTGGCGCGGCCGCACCGCAATCACGGCCCCGTGTGGAA-3‘
Leptin-fw	5‘-CTCGAGAGCCCCTCAGGGATCTTGCATTC-3‘
Leptin-rv	5‘-GCGGCCGCAACCCTTCAAGGTCCCTTCAG-3‘
PPARγ-fw	5‘-CTCGAGAGCGAGCCCAAGTTTGAGTTTGC-3‘
PPARγ-rv	5‘-GCGGCCGCAAGGTGTCAGATTTTCCCTCA-3‘
RASD1-fw	5‘-GGTGGCGCACTCGAGCCGTGCCCGCTTGAGGGTCAGGA-3‘
RASD1-rv	5‘-GGTGGCGCGGCCGCACCGTAAAGACCTCACACACACAC-3‘
TNFα-fw	5‘-CTCGAGAGCGGGCCTACAGCTTTGATCCC-3‘
TNFα-rv	5‘-GCGGCCGCAGGAGCAGAGGCTCAGCAATG-3‘
primer for mutagenesis	
ADIPOQ-fw mt1	5‘-CCCTACACTACTCTACTTCC-3‘
ADIPOQ-rv mt1	5‘-GGAAGTAGAGTAGTGTAGGG-3‘
AR-fw mt1	5‘-AACTCTACACTACTCCTCTG-3‘
AR-rv mt1	5‘-CAGAGGAGTAGTGTAGAGTT-3‘
ARHGEF12-fw mt1	5‘-ATTTTCGCACTGATGGTGCCAAAGG-3‘
ARHGEF12-rv mt1	5‘-CCTTTGGCACCATCAGTGCGAAAAT-3‘
ARHGEF12-fw mt2	5‘-GGCTGTATGTTTCTGGCACAAAATATTGGTCATC-3‘
ARHGEF12-rv mt2	5‘-GATGACCAATATTTTGTGCCAGAAACATACAGCC-3‘
Leptin-fw mt1	5‘-CCCTGCTCGCACTTTGTAAC-3‘
Leptin-rv mt1	5‘-GTTACAAAGTGCGAGCAGGG-3‘
PPARγ-fw mt1	5‘-TTCTTCCAGTCGCACTATTC-3‘
PPARγ-rv mt1	5‘-GAATAGTGCGACTGGAAGAA-3‘
RASD1-fw mt1	5‘-CAAAACTCGCACTTTAACGGTAGTTCC-3‘
RSASD1-rv mt1	5‘-GGAACTACCGTTAAAGTGCGAGTTTTG-3‘
RASD1-fw mt2	5‘-GGACACGCACGAAACCTTAC-3‘
RASD1-rv mt2	5‘-GTAAGGTTTCGTGCGTGTCC-3‘
TNFα-fw mt1	5‘-GCCAGCTCCCTCTATTTATGTTCGCACTTG-3‘
TNFα-rv mt1	5‘-CAAGTGCGAACATAAATAGAGGGAGCTGGC-3‘

### Statistical analyses

All experiments were performed at least three times. MicroRNA expressions measured by qRT-PCR and luminescence values gained from the luciferase reporter assay experiments were visualized as relative mean ± standard deviation. All results were analysed by Student’s t-test, in which p < 0.05 was considered as significant.

## Results

### miR-130a and miR-301 expression during adipogenesis

**Figure 1.**
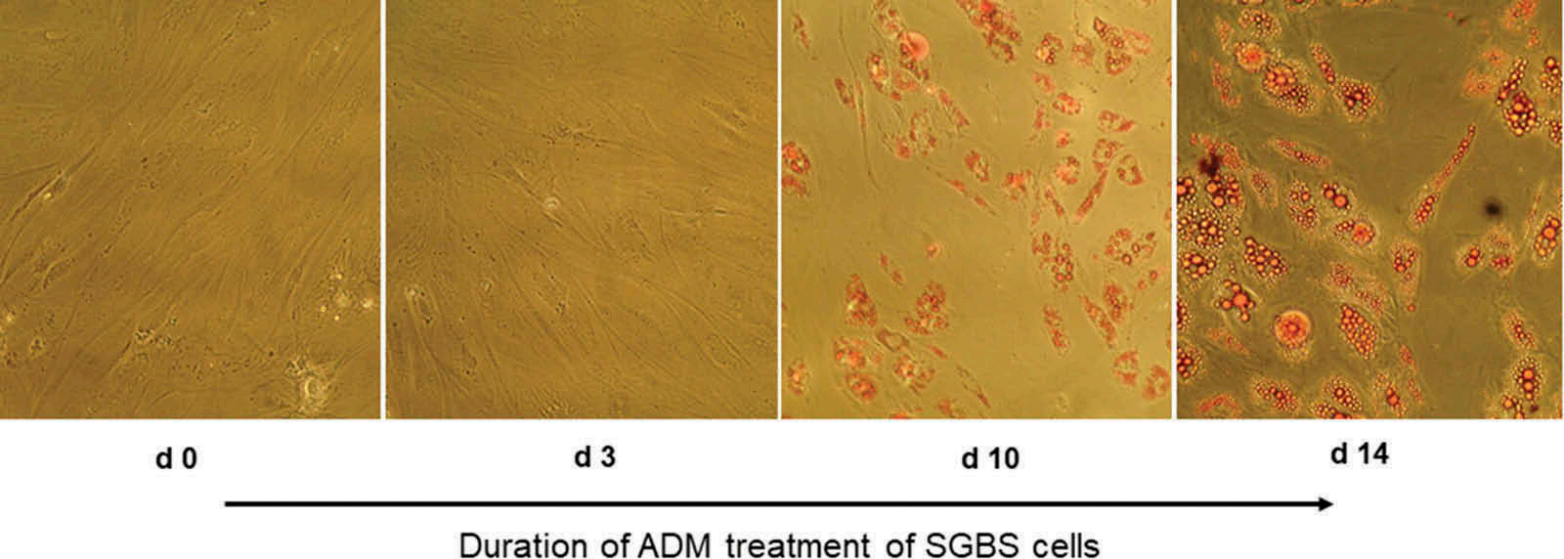
OilRed O staining of SGBS preadipocytes on d0, d3, d10 and d14 of adipogenic differentiation. Cells were treated with adipogenic differentiation medium and visualized under magnification x100

**Figure 2.**
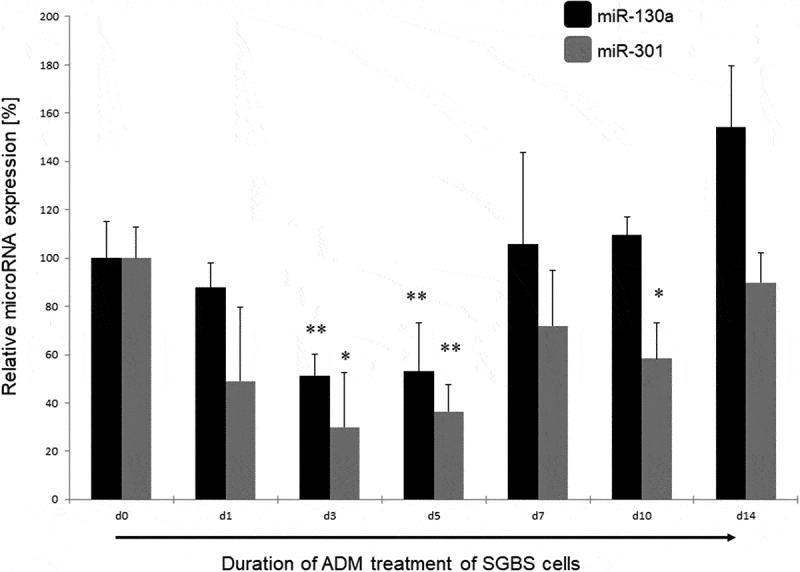
miR-130a and miR-301 expression during adipogenic differentiation of SGBS cells. Legend: black bars: miR-130a expression, grey bars: miR-301 expression. Respective d0 values are set for 100%. Abbreviations: d = day; *p < 0.05; **p < 0.01; n = 3, Student’s t-test, mean ± standard deviation

The adipogenic differentiation of the SGBS cells used under treatment with adipogenic differentiation medium (ADM) was verified by OilRed O staining (see Figure 1). We measured the expression of miR-130a and miR-301 via qPCR with specific TaqMan primers during the time course of adipogenesis. Measurements were performed at the initiation of the adipogenic differentiation via ADM (d0) as control point, and on day 1 (d1), day 3 (d3), day 5 (d5) – corresponding to the determination phase – and day 7 (d7), day 10 (d10) and day 14 (d14) – corresponding to the terminal differentiation phase. MiR-130a expression was significantly reduced on about 50% at d3 and d5 of the determination phase of the adipogenic differentiation (p = 0.005 and p = 0.007, respectively; see Figure 2) in comparison to d0. However, miR-130a expression did not exhibit significant differences to d0 in the terminal differentiation phase (d7, d10 and d14). Analogous, also miR-301 was significantly downregulated to 30 – 36% during d3 and d5 of adipogenesis in comparison to d0 (p = 0.032 and p = 0.009, respectively, see Figure 2). Subsequently, miR-301 remained lower expressed during the terminal differentiation phase on around 58% (d10, p = 0.04) to 72% (d7). Therefore, we propose a regulative role for both microRNAs during the determination phase of the adipogenic differentiation process.

### miR-130a and miR-301 expression in response to androgen stimulation

**Figure 3.**
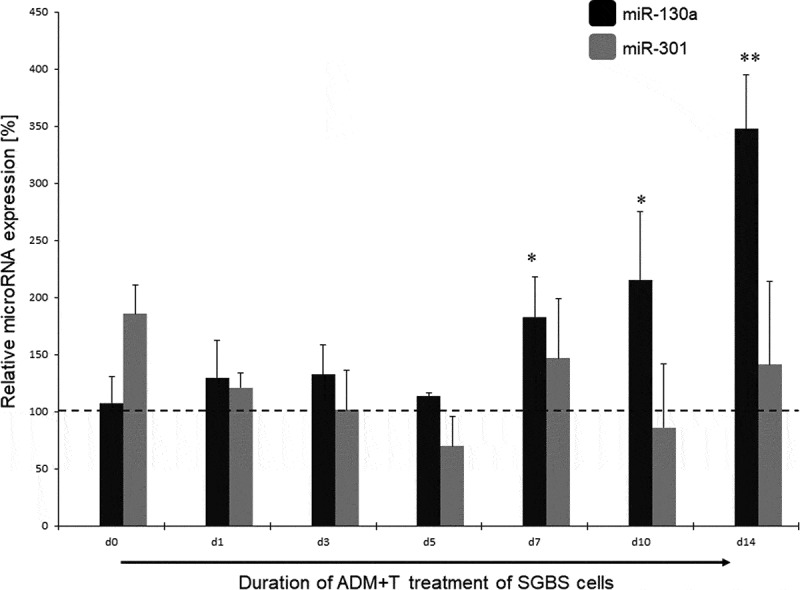
miR-130a and miR-301 expression during adipogenic differentiation of SGBS cells. Legend: black bars: miR-130a expression under ADM + testosterone treatment, grey bars: miR-301 expression under ADM + testosterone treatment. Dotted line represents the DMSO control individually set for 100%. Abbreviations: d = day; *p < 0.05; **p < 0.01; n = 3, Student’s t-test, mean ± standard deviation

**Figure 4.**
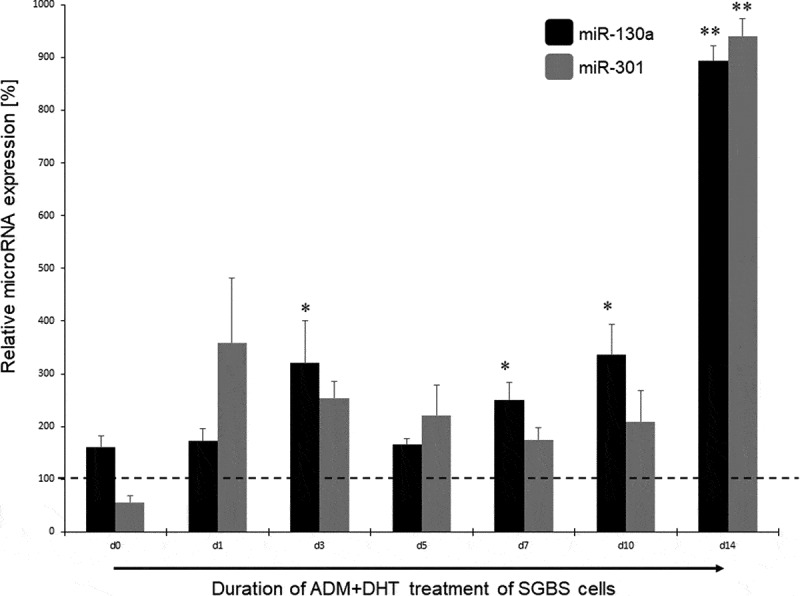
miR-130a and miR-301 expression during adipogenic differentiation of SGBS cells. Legend: black bars: miR-130a expression under ADM + DHT treatment, grey bars: miR-301 expression under ADM + DHT treatment. Dotted line represents the DMSO control individually set for 100%. Abbreviations: d = day; *p < 0.05; **p < 0.01; n = 3, Student’s t-test, mean ± standard deviation

In the next step, we analysed the miR-130a and miR-301 expression during androgen-induced adipogenesis inhibition. In comparison to a DMSO treated control set to 100% for each individual time point, miR-130a expression rose significantly during d7 – d14 under stimulation with testosterone (180 – 350%, p = 0.03–0.007, respectively; see Figure 3) or DHT (250 – 900%, p = 0.02–0.00004, respectively, see Figure 4). On the other hand, no significant regulation of miR-301 was detectable in the testosterone-stimulated SGBS cells (see Figure 3), while under DHT stimulation only at the endpoint of adipogenic differentiation (d14) a significant increase in miR-301 on 940% in comparison to the DMSO treated control was observed. Therefore, we propose miR-130a as an androgen-regulated microRNA directly affecting the course of adipogenesis by changing cell fate through regulating adipogenesis-related genes in the differentiation process.

### miR-130a target gene analysis

**Figure 5.**
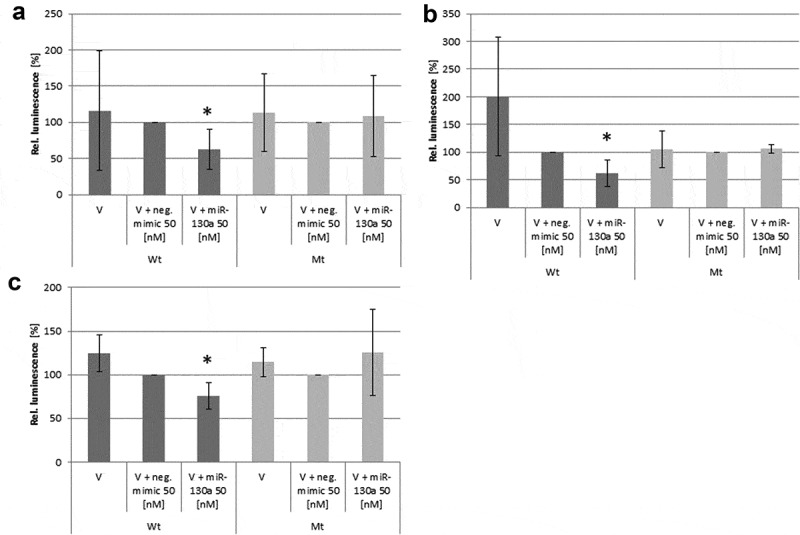
Luciferase reporter assays on the regulation of several target genes by miR-130a. (a): Androgen receptor (AR)-3ʹUTR; (b): Adiponectin (ADIPOQ)-3ʹUTR; (c): Tumour necrosis factor alpha (TNFα)-3ʹUTR. Abbreviations: V = vector; wt = wildtype sequence; mt = mutated sequence; n = 6; *p < 0.05, Student’s t-test

Subsequently, we evaluated by luciferase reporter assay, whether the differentiation-related genes androgen receptor (AR), adiponectin (ADIPOPQ), PPARγ, Tumour necrosis factor alpha (TNFα), Leptin, ARHGEF-12 and Ras, dexamethasone-induced 1 (RASD1) are *in vitro* target genes of miR-130a and miR-301. All mRNAs were detected as putative targets of these microRNAs in a comparative database analysis (see Table 1) with different certainties. As we detected only miR-130a to be androgen-regulated during adipogenic differentiation, we primarily tested the translation inhibitory capacity of this microRNA on the *in silico* identified adipogenesis-related target genes in luciferase reporter assays. Interestingly, only the AR (p = 0.0002; Student’s t-test; see Figure 5(a)), ADIPOQ (p = 0.03; Student’s t-test; see Figure 5(b)) and TNFα (p = 0.02; Student’s t-test; see Figure 5(c)) exhibited negative regulation of their respective wt 3ʹUTR by miR-130a. The other putative target genes exhibited no significant changes in luciferase activity due to miR-130a cotransfection (data not shown). Interestingly, though being previously described as miR-130a target gene, PPARγ exhibited no significant regulation by miR-130a in our luciferase reporter assay experiments (p = 0.07; Student’s t-test), but showed a trend towards regulation. Therefore, we propose AR, ADIPOQ and TNFα as adipogenesis-relevant target genes of miR-130a and *bona fide* target genes of miR-301.

## Discussion

In this study, we analysed the expression of miR-130a and miR-301 during adipogenesis of human SGBS preadipocytes as well as the impact of androgen-induced adipogenesis inhibition on these microRNAs. Additionally, we tested the inhibitory effect of miR-130a by direct binding to the 3ʹUTR of several adipogenesis-related genes via luciferase reporter assay.

Firstly, we were able to demonstrate a significant downregulation of miR-130a and miR-301 in the initial differentiation phase of the adipogenesis of human SGBS cells. Recently, Seenprachawong and colleagues as well analysed the expression of miR-130a and miR-27b during osteogenesis and adipogenesis, and concordantly identified miR-130a to be significantly downregulated at day 3 of adipogenesis, while on later time points no significant regulation of this microRNA could be detected [31]. Vimalraj and Selvamurugan demonstrated that a significant upregulation of miR-130a, among others, was specific for differentiated osteoblast after 14 d in comparison to undifferentiated hMSCs [25]. Recently, Lin and colleagues demonstrated that miR-130a overexpression inhibits the adipogenic differentiation of murine bone marrow derived mesenchymal stem cells, while promoting the osteoblastic differentiation [32]. These results point towards an interference of miR-130a with early phases of adipogenesis. In line with this hypothesis, miR-130a upregulation was also shown to activate Wnt signalling in hepatocellular carcinoma cells by targeting RUNX3 expression [33]. Additionally, miR-130a was demonstrated to be upregulated by hypoxia in gastric cells, also targeting RUNX3 and therefore increasing - in combination with miR-495 – cell proliferation while lowering apoptosis [34]. These interactions may also be exerted via the Wnt/β-Catenin signalling pathway, as this axis was demonstrated to be active in gastric cancer cells [35]. Together, these data point towards a regulative role of miR-130a during the early phase of several differentiation processes.

Next, we observed a significant upregulation of miR-130a, but not miR-301, in response to prolonged androgen stimulation. While there are several well-studied androgen-regulated microRNAs, including miR-21, miR-125b, miR-148a, miR-106b and miR-29a/b, in prostate carcinoma (reviewed in [36]), little is known about the interaction of miR-130a with the androgen signalling pathway in other cell types. Interestingly, miR-130a is downregulated in several prostate carcinoma cell lines, including the androgen-responsive LNCaP, in comparison to normal prostate epithelium [37]. Moreover, Boll and colleagues demonstrated that reconstitution of miR-130a expression in LNCaP changed the cellular phenotype comparable to AR knockdown, and that miR-130a in combination with miR-205 suppresses AR signalling significantly [37]. Therefore, the connection of androgen receptor signalling and miR-130a/miR-301 expression should be studied in further detail.

We established AR, ADIPOQ and TNFα as miR-130a target genes in luciferase reporter assays. While TNFα regulation via miR-130a already has been shown in cervical cancer cells by EGFP reporter assays and Western blotting [38], only indirect hints on a miR-130a regulation of AR existed so far in prostate carcinoma cell lines [37] and mouse sertoli cells [39]. Another putative target gene of miR-130a – PPARγ – exhibited only a trend towards significance in our experiments for a regulation by miR-130a. However, PPARγ regulation by miR-130a has been demonstrated in various organisms including humans [31,32,40–42]. To the best of our knowledge our results are the first detecting AR and ADIPOQ as miR-130a target genes in the context of adipogenesis.

In the light of growing obesity prevalence across the world, in association with the corresponding metabolic health aspects, there is a special interest in the factors regulating adipogenesis and therefore laying the ground for an increase in body fat mass. A comprehensive study on circulating microRNAs in the serum of patients with different cardiovascular risk factors in comparison to healthy controls exhibited significant downregulation of miR-130a in the serum of patients with diabetes mellitus type 2 or hypercholesterolemia, but significant upregulation in patients with metabolic syndrome or hypertension [43], indicating that miR-130a may play also a role in the differentiation processes of other cell types. Furthermore, in acute ischaemic stroke patients circulating miR-130a levels were correlated with reduced inflammatory cytokine levels, therefore being associated with a lower disease risk and severity [44]. Altogether, these data point towards a key role of a dysregulated miR-130a expression and action in the genesis and progression of metabolic diseases yet warranting further analysis.

In conclusion, we demonstrated a significant downregulation of miR-130a and miR-301 in the early phase of human SGBS preadipocyte differentiation. Conversely, miR-130a expression was significantly upregulated by androgen stimulation during adipogenesis, while androgen treatment had no significant effect on miR-301 expression. AR, ADIPOQ and TNFα were validated as adipogenesis-related target genes of miR-130a in *in vitro* luciferase assays. Therefore, we propose miR-130a as androgen-induced microRNA involved in adipogenesis inhibition via the fine-regulation of adipocytic gene expression.
